# Editorial: Advances in lung ultrasound: from child to adulthood diseases

**DOI:** 10.3389/fmed.2024.1392319

**Published:** 2024-03-20

**Authors:** Ioana Mihaiela Ciuca, Luigi Vetrugno

**Affiliations:** ^1^Department of Pediatrics “Victor Babes”, University of Medicine Timisoara, Timişoara, Romania; ^2^Department of Medical, Oral and Biotechnological Sciences, University of Chieti-Pescara, Chieti, Italy; ^3^Department of Anesthesiology, Critical Care Medicine and Emergency, SS. Annunziata Hospital, Chieti, Italy

**Keywords:** lung ultrasound, adult, children, intensive care, neonatal intensive care

In this first Research Topic dedicated to the *Advances in Lung Ultrasound: From Child to Adulthood Diseases: Part I*, we received seven articles exploring important and varying aspects of the field.

In their quasi-physiological study “*Ultrasound assessment of the respiratory system using diaphragm motion-volume indices*”, Boussuges et al. matched ultrasounds of the diaphragm with the respiratory system in 122 subjects (51 women and 71 men) with normal pulmonary function. They compared this measurement to patients with one paralyzed hemidiaphragm. They discovered that an increase in the neural tract develops on the healthy diaphragm side to compensate for decreased contralateral diaphragm paralysis. This is a very important update when considering the process of weaning critically ill patients. We suggest this review for those who are not experts in this field (1).

In a second article, “*Pleural clinic: where thoracic ultrasound meets respiratory medicine*”, Tinè et al. described the force of a Pleural Clinic “team” at the University of Padua that began by treating outside patients but shortly became a point of reference for inpatients and other colleagues. Of particular interest in this article was the development of point-of-care ultrasound (POCUS) protocols for the global assessment of COVID-19 patients, especially those in critical conditions. Thoracic ultrasound was effectively integrated to evaluate all the possible consequences of SARS-CoV-2 infection, including cardiac dysfunction, parenchymal involvement, and thrombotic complications.

In this organization, thoracic ultrasound offered a safe and effective tool to identify the problem, suggest the best setting to manage it, and guide diagnostic/therapeutic procedures. The results of this group are entirely in line with the recent British Thoracic Society Clinical Statement on pleural procedures guidelines (2).

Another important study is the multicenter study on the accuracy of lung ultrasound in the diagnosis and monitoring of respiratory sequelae in medium- and long-term patients with COVID-19 by Ramos Hernández et al., which contained two central aspects. First was that lung ultrasound could be implemented as a first-line procedure in evaluating interstitial lung sequelae after COVID-19 pneumonia. Second and, in our opinion, most importantly, this study showed the real power of lung ultrasound when also considering the economic and environmental impacts (reducing ionized radiation emission and cost) over chest X-ray and CT scans. Lung ultrasound has important applications in the pediatric population as a reliable, non-irradiating, and easy to use method for several diseases including acute neonatal respiratory distress syndromes (3). The article written by Huang et al., “*The value of lung ultrasound score in neonatal respiratory distress syndrome: a prospective diagnostic cohort study*”, evaluated the relation between LUS score and outcome-predicting parameters like oxygenation and respiratory indexes and sequential organ failure assessment score; they demonstrated a significant correlation of LUS score with the severity index. The association of imagistic LUS score and clinical scores had a significant predictive value in evaluating the severity and prognosis of neonatal distress syndrome, demonstrating the significant role of the LUS to asses lung changes in a very complex population like infants and children (4). In the article on bronchial obstruction in patients with osteogenesis imperfecta (OI), entitled “*Bronchial obstruction in osteogenesis imperfecta can be detected by forced oscillation technique*”, the authors demonstrate that forced oscillation technique can detect bronchial obstruction in patients with a rare pathology (Storoni et al.). The difficulty in evaluating these patients is due to a number of factors; for example, spirometry is a challenge as it requires forced breathing. The study‘s outcome revealed that FOT is a consistent alternative technique for lung function measuring in the study population, being able to assess expiratory flow limitation as well as lung compliance changes. The importance of the study resides in functional evaluation of bone- and lung-affecting chronic disease, especially significant as lung disease is the most important outcome predictor for this pathology (5), and it is imperative to envisage the changes in lung structure and function (6).

In the article “*Thoracic ultrasound combined with low-dose computed tomography may represent useful screening strategy in highly exposed population in the industrial city of Taranto*”, 706 subjects were evaluated for the risk of pollution in an industrialized region of Italy (Quarato et al.). In this study, the lung ultrasound screening protocol showed that LUS could act as a complementary imaging technique in assessing patients who could benefit from a second-low-dose high-resolution computed tomography (HRCT) scan. The study was conducted on high-risk, asymptomatic patients with lung cancer and interstitial lung abnormalities. LUS had an important diagnostic accuracy of 88.6% and an increased sensitivity of 95.3%, compared to HRCT as gold standard, in detecting pleuro-pulmonary anomalies such as interstitial lung abnormalities, subpleural, or pulmonary nodules. The study demonstrated ultrasound as a potential screening tool for lung cancer and fibrosis in a population highly exposed to pollution who could benefit from a second chest HRCT. It is a significant achievement for lung ultrasound to be able to identify patients at risk for lung cancer.

With so many rapid diagnostic image modalities available for respiratory disease (chest X-ray, lung ultrasound, electrical impedance tomography (EIT), and computed tomography (CT) scan), we can say, metaphorically, that those who used lung ultrasounds before COVID-19 are more likely to flourish. Although we know the limitations of lung ultrasound, only time can give us a definitive conclusion (7, 8).

We hope that the new Research Topic “*Advances in Lung Ultrasound: From Child to Adulthood Diseases: Part II*” can help to advance this field (https://www.frontiersin.org/research-topics/63026/advances-in-lung-ultrasound-from-child-to-adulthood-diseases—volume-ii/overview).

## Author contributions

IC: Writing – review & editing, Writing – original draft, Supervision, Conceptualization. LV: Writing – review & editing, Writing – original draft, Validation, Supervision, Conceptualization.
